# A Case of Unrecognized Intrathoracic Placement of a Subclavian Central Venous Catheter in a Patient with Large Traumatic Hemothorax

**DOI:** 10.1155/2015/382624

**Published:** 2015-08-11

**Authors:** Dina Wallin, Alicia R. Privette, Andre R. Campbell, Julin F. Tang

**Affiliations:** ^1^UT Austin Pediatric Emergency Medicine Fellowship, Dell Children's Medical Center, 4900 Mueller Boulevard, Austin, TX 78723, USA; ^2^Department of Surgery, Medical University of South Carolina, 96 Jonathan Lucas Street, Charleston, SC 29425, USA; ^3^Division of General Surgery, San Francisco General Hospital, Campus Box 0807, San Francisco, CA 94143-0807, USA; ^4^Department of Anesthesia and Critical Care Medicine, San Francisco General Hospital, 1001 Potrero Avenue, SFGH 5, San Francisco, CA 94110, USA

## Abstract

Traditional recommendations suggest placement of a subclavian central venous catheter (CVC) ipsilateral to a known pneumothorax to minimize risk of bilateral pneumothorax. We present the case of a 65-year-old male with a right hemopneumothorax who was found to have intrathoracic placement of his right subclavian CVC at thoracotomy despite successful aspiration of blood and transduction of central venous pressure (CVP). We thus recommend extreme caution with the interpretation of CVC placement by blood aspiration and CVP measurement alone in patients with large volume ipsilateral hemothorax.

## 1. Introduction

In a trauma patient with pelvic fractures and cervical spine immobilization, the subclavian vessels are the most viable option for central venous catheterization. Known risks of subclavian catheterization include pneumothorax, arterial puncture, hematoma, and incorrect placement, most commonly in the ipsilateral internal jugular vein but described in locations from the thoracic cavity [1, 2] to the aorta [3]. Given the procedural risk of pneumothorax, it is generally recommended that when placing a subclavian central venous catheter (CVC) in the trauma patient with a known pneumothorax the CVC be placed ipsilaterally [4]. We present a case of unrecognized intrathoracic placement of a right subclavian CVC in a patient with a large ipsilateral hemothorax.

## 2. Case Presentation

A 65-year-old male presented to the emergency department (ED) following a motorcycle collision. In the ED, his airway was intact, he had decreased breath sounds on the right chest, and his initial systolic blood pressure (SBP) was 130 mmHg. A right hemothorax was seen on conventional radiography; thus, a right tube thoracostomy was performed, with immediate drainage of approximately 500 cc of blood. He was then transported to the intensive care unit (ICU).

**Figure 1:**
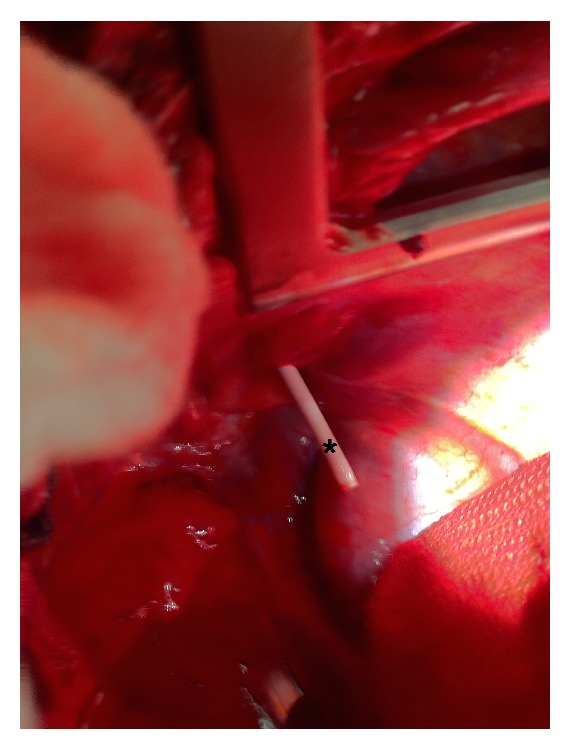
Subclavian central venous catheter (asterisk) in thorax.

On arrival to the ICU, the patient's SBP was in the 80 s mmHg and his right chest tube had drained nearly one liter of blood. A right subclavian CVC was placed using landmark guidance, with easy aspiration of dark red, nonpulsatile blood and a central venous pressure (CVP) reading of 3 cm H_2_O. Chest tube output had slowed dramatically and the SBP was stable at 90 mmHg. A packed red blood cell transfusion was initiated through his CVC. Over the next hour, he had over one liter of bloody chest tube output and his blood pressure began to drop; he was then transported to the operating room. On thoracotomy, the right subclavian CVC was noted to have perforated the subclavian vein and was instead located in the right hemithorax (Figure 1).

## 3. Discussion

Traditional teaching recommends placing subclavian CVC ipsilateral to known pneumothorax; thus, in this patient with a right hemopneumothorax, the decision was made to insert a right subclavian CVC. Although blood was easily aspirated from the catheter lumen, this aspirate was likely from intrathoracic contents. Hohlrieder et al. [2] describe a similar case of transpleural placement of a subclavian CVC, recommending aspiration of a greater volume of blood than the intraluminal catheter volume; this would not have helped in our case, given the patient's large volume of intrathoracic blood. Sanders et al. [5] describe an iatrogenic tension hemopneumothorax in a patient with isolated extrathoracic trauma after inserting a subclavian CVC into the thoracic space and pressure transfusing blood products into the previously uninjured thorax. While ultrasound-guided subclavian venous catheterization has been reported to decrease mechanical complications such as pneumothorax, hemothorax, and arterial puncture, real-time sonography does not appear to decrease the rate of catheter malposition [6] and might not have helped this patient. Our case is the first to describe intrathoracic placement of a subclavian CVC in a patient with thoracic trauma, unrecognized until thoracotomy. We suggest that, in patients with large volume hemothorax, providers exercise caution in utilizing blood aspiration and CVP measurement to assume appropriate intravascular placement of a subclavian CVC. Prior to infusion of potentially dangerous substances or of large volumes, correct intravascular position should be confirmed by conventional radiography.
